# Effect of nutritional education on anthropometric deficits among pre-school aged children in south West Ethiopia: quasi-experimental study

**DOI:** 10.1186/s13052-022-01201-0

**Published:** 2022-01-15

**Authors:** Kebebe Bidira, Dessalegn Tamiru, Tefera Belachew

**Affiliations:** Department of Nutrition and Dietetics, Institute of Health, Jimma University, Jimma, Ethiopia

**Keywords:** Anthropometric deficits, Nutrition education, Preschool children, Ethiopia

## Abstract

**Background:**

Under-nutrition is a global problem and one of the most serious public health issues. Globally, 156 million under-five children were stunted, and 50 million were wasted in 2016. Malnutrition among preschool-age children is caused by low socioeconomic status, food insecurity, poor feeding practices, and infectious diseases. This intervention aimed to evaluate the effect of nutrition education delivered through trained health professionals in improving the nutritional status of preschool -aged children.

**Methods:**

A quasi-experimental design among 588 preschool –aged children was used. A multistage sampling technique followed by a systematic random sampling technique was used to identify caregivers with preschool-aged children. Structured questionnaires were used to collect data. The baseline difference in demographic and socioeconomic characteristics between the two groups was examined using a chi-square test and an independent sample t-test was used to determine the mean difference in under-nutrition between the intervention and control groups. Generalized estimating equations (GEE) were used to determine the change in the difference in outcome between the intervention and control groups as well as the association of predictors with under-nutrition in children. The Adjusted odds ratio (AOR) with the corresponding 95% confidence intervals was reported to show the strength of the association. Variables with a *p*-value of less than 0.05 were considered statistically significant in multivariable analysis.

**Results:**

In this study, the nutritional status of preschool age children was significantly associated with nutrition education intervention [AOR = 0.566, 95% CI: (0.347, 0.923)], place of delivery [AOR = 0.724, 95% CI: (0.551, 0.951)], ARI in the last 2 weeks [AOR = 1.823, 95% CI: (1.226, 2.710)], source of drinking water [AOR = 0.624, 95% CI: (0.484, 0.805)] and household food security [AOR = 1.311, 95% CI: (1.030, 1.669)] .

**Conclusions:**

Findings of this study showed that nutrition education can effectively reduce the magnitude of under-nutrition among preschool children. Under-nutrition was e significantly associated with nutritional education, place of delivery, ARI in the last 2 weeks, source of drinking water, and food security. Therefore, both government and non-government should consider the impacts of nutrition education to alleviate under-nutrition and improve the health status of preschool-age children.

## Background

Under-nutrition is a global problem and one of the most serious public health issues, obstructing cognitive and physical growth and contributing to child morbidity and mortality [1]. Globally, 156 million children under the age of five were stunted, 50 million were wasted, and 42 million were overweight in 2016 [2]. In terms of preschoolers, a 2012 report projected that 183 million are underweight, 226 million are stunted, and 67 million are wasted in developing countries [3]. According to a World Bank report, Asia and Africa have the highest rates of stunted and wasted children, with Asia responsible for 56% of stunted children and Africa for 37% of stunted children and 25% of wasted children. According to the study, the global trend in stunting, wasting, and underweight is decreasing, but at a slow rate [2].

In 2016, 38% of Ethiopian children under the age of five were stunted, 10% were wasted, and 24% were underweight [4]. Every year, an estimated 350,000 children in Ethiopia die, mostly from preventable and treatable diseases, as well as infectious diseases aggravated by malnutrition [5]. Preschool aged children need proper care because they not only have special needs, but it also provides a basis for all children’s growth and development. Malnutrition in preschool children is caused by a complex interplay of factors such as birth weight, household food access, drinking water availability and use, sanitation, and child and maternal care [6].

Anthropometric indices such as height for age, weight for age, and weight for height are commonly used to assess under-nutrition in preschool-aged children. Low height for age (stunting) is a sign of chronic malnutrition, as evidenced by skeletal growth problems. Low weight for age (wasting) is a sign of acute malnutrition, which results in the loss of both lean and fat mass. Low weight for age (underweight) is reflective of both acute and chronic malnutrition [6–8]. The Composite index of anthropometric failure (CIAF) was introduced by Svedberg et al., 2000 and modified by Nandy et al. in 2005 to estimate the burden of under-nutritional, since none of the three conventional indices are able to provide the overall prevalence of under-nutrition in the population. This is due to the fact that these indices overlap, which means that the same child may show signs of two or more anthropometric failures at the same time [6, 9].

Inadequate dietary intake, illness/disease, household food insecurity, inadequate child care and feeding practices, unhealthy household and surrounding conditions, and inaccessible and frequently inadequate health care are among the causes of under-five child malnutrition, according to UNICEF’s conceptual framework [10]. According to studies, malnutrition among preschoolers is related to low socioeconomic status, food insecurity, inadequate feeding practices, and infectious diseases [11]. Improper feeding practices are often related to under-nutrition in children under the age of five, according to the World Health Organization (WHO) [12]. According to the World Bank, inadequate diets in terms of diversity, quality, and quantity, as well as disease, are the leading causes of child malnutrition [2].

The other research, which was conducted in Malawi, found that low dietary quality and quantity, as well as improper feeding practices, can lead to under-nutrition and poor nutritional status in early childhood, which is related to growth failure [13]. In Ethiopia, children’s malnutrition is still a major problem. Many studies have been conducted among children under the age of five to determine the prevalence of under-nutrition and the factors that contribute to it, but few studies have been conducted among preschool-aged children. However, instead of CIAF, conventional indices were used in all of these studies across the world.

All preschool-aged school children require a comprehensive nutrition education intervention program that encourages healthy eating and active living so that they can have a healthy lifestyle, have good nutritional status, have better cognitive function, and have a decent quality of life. The evidence from the identified studies suggests that community-based nutrition education improves the nutrition status of under-five children in developing countries [14]. We hypothesized that nutrition education offered by trained health professionals and health extension workers is more effective than routine health and nutrition care in reducing preschooler undernourishment. The present nutrition education intervention aimed to evaluate the effectiveness of nutrition education intervention delivered through trained health profession in improving the nutritional status of preschool children.

## Methods and materials

### Study design and setting

Ilu Abba Bor is a zone in the Oromia regional state, 600 km from the country’s capital, Addis Ababa. The Zone is divided into 14 districts, one administrative town, and 13 rural districts, with a total population of 934,783 people, including 153,585 children under the age of five and 100,209 children aged two to five years.

It stretches from 70 27 ‘40 “N to 90 02’ 10” N latitude and 340 52′12 “E to 410 34’55” longitude in the western part of the country. The climate is divided into three main zones: temperate, humid, rainy, and dry arid. Rainfall occurs twice a year which ranges from 2400 mm to 100 mm. in most highland areas of Ilu Abba Bora, the highest mean annual temperature ranges from 26 °C to 10.6 °C.

### Study design

A quasi-experimental design with pre-test-post-test and control groups was used from May 15, 2019 to February 15, 2020 to assess the effectiveness of community-based nutritional education on nutritional status among preschool-aged children in 22 rural kebeles.

### Sample size and sampling technique

**Fig. 1:**
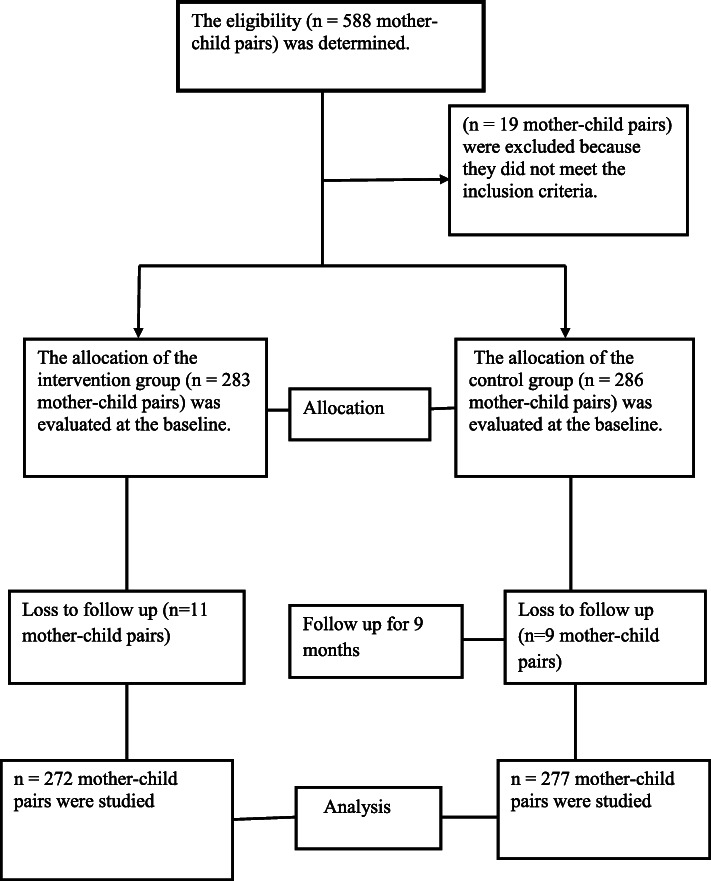
shows a flow diagram of the research process

The sample size was calculated using a G-Power model with the following assumptions: 52.5% expected prevalence of stunting among children aged 24 to 59 months, 2.07 odds ratio of acute respiratory tract infection [15], 80% power, and a 5% margin of error. Then, a design effect of 2 and a 15% non-response rate were taken into consideration and the final sample size was 588 (Fig. 1).

A multi stage sampling technique followed by systematic random sampling technique was used to identify caregivers with preschool aged children. In the first stage, four districts were chosen purposively from the 14 districts. Due to their similarity in terms of access to health care, water, and other services, two neighboring districts from the selected districts were chosen as intervention and the other two adjacent districts as control groups to prevent information contamination. Then, at random, two districts were allocated to the intervention group and the other two districts to the control group. Eleven kebeles (Ethiopia’s smallest administrative units) were chosen at random from each community. Following the selection of kebeles, pre-school aged children (2–5 years) were identified and registered in each chosen kebele. Households with children aged 2 to 5 years were given a code, which was used as a sampling frame. Following the registration of preschool-aged children, a proportional allocation was carried out, and a sample was drawn from both the intervention and control groups using a systematic random sampling technique. If the caregivers/households had more than one child, one was selected at random using the lottery method.

### Nutritional intervention strategy

Following the completion of the baseline data collection, nutrition education was implemented for a total of 9 months in all intervention groups. A nutrition education package was developed using family dietary guidelines [16] and other relevant nutritional education intervention modules and topics were adopted and modified as local situations from literature [17, 18] for community-based nutrition intervention as well as using the findings of a base line survey. It is easy, interactive, and user-friendly for all trained professionals, health extension workers, and caregivers/mothers to use in implementing nutrition interventions for mothers/caregivers of preschool-aged children at the community level.

The Nutrition educational package consists of three educational modules and a number of supporting educational materials. The education modules addressed three main aspects: healthy diet awareness, nutrition, and hygiene, with a total of ten topics, namely: Enjoying a variety of foods, Feeding toddlers and preschoolers aged 2 to 5 years; Make starchy foods the base of the majority of your meals. Consuming a variety of vegetables and fruits on a daily basis, regularly consume dry beans, split peas, lentils, and soya. Chicken, fish, meat, milk, or eggs can be eaten daily; Use salt sparingly. Consume fat sparingly. Use food and drinks containing sugar sparingly and not between meals; this keeps food safe and clean.

The caregivers/mothers gathered in a school, health postl, or health center that was convenient for them and comfortable for the group. Nutrition education was provided to eleven kebele caregivers/mothers by trained health professionals. One trained health professional and one health extension worker were assigned to each kebele, implying that health extension workers acted as assistants and facilitators. Over the course of five months, ten sessions were held every two weeks for approximately 30–45 min, with two sessions held in the seventh month as a refresher. The topic of the day was introduced to the group of caregivers on the day of the presentation. Following that, specific questions about the topic were asked to assess prior knowledge and encourage discussion. The caregivers were evaluated at the end of the session to see how well they understood the information.

The communication mechanism is determined by the context, cultural preferences, and the way in which individuals normally receive and obtain information. A set of educational materials, including group discussions, lectures, role-plays, active participation, and demonstrations, was used to teach mothers knowledge and skills about child feeding. The group discussion method was used more in this study because it is an excellent way to encourage interaction between group members and it allows for more participation.

### Process evaluation

Monitoring activities and process evaluation were used to improve and track compliance with the intervention. The principal investigator and supervisors visited each household to monitor the implementation of the intervention and discuss the activities with the mothers/caregivers. The principal investigator made many visits to the field. Investigators and supervisors have met on a regular basis to review the project’s progress and discuss any necessary changes. The goal of the process evaluation was to document the intervention’s implementation process in order to see if the activities were carried out as planned, determine the degree to which the intervention reached children and mothers/caregivers; evaluate the extent to which the intervention reached children and mothers/caregivers; determine the extent to which intervention components are exposed to target children and caregivers; investigate contextual factors that may influence intervention effectiveness, and provide information that can help in the interpretation of outcome indicators and also be used to monitor the attendance of mothers/caregivers during nutritional education sessions.

### Data collection instruments and procedure

A baseline analysis of 569 caregivers or mothers with preschool-aged children was conducted at the time of enrollment. Trained data collectors used a structured interviewer questionnaire to interview all caregivers/mothers at the start and end of the study. The questionnaire was written in English first, then translated into the local language (Afan Oromo), and finally back to English to ensure consistency and quality of the data. A pre-test of the questionnaire was conducted outside the study area on 5% of the total samples to determine the acceptability and applicability of the tools and procedures before the real data collection. Over the course of three days, all data collectors and supervisors received extensive training.

To avoid within-examiner error, all anthropometric measurements were taken by investigators, supervisors, and data collectors. Before the measurement, the weight scale was set to zero and placed on a level surface. The age of the child was calculated using the child’s date of birth and the date of the interview. Where the exact date of birth was not registered or unknown, the caregiver was asked to guess based on local events. By subtracting the child’s date of birth from the date of data collection, the child’s age was calculated. Each questionnaire was monitored and double-checked by a principal investigator and professional supervisors for completeness, irregularities, inconsistencies, and out-of-the-ordinary responses, making immediate corrections as needed. During data entry, computer frequencies were used to check for missing variables, outliers, and other errors.

The dietary diversity score was assessed using the repeated 24-h dietary recall method, which was taken three, times (two weekdays and one weekend). Every day of the week was described, including fasting and feasting days. The children’s caregivers were asked to remember everything their children ate or drank during the 24-h period of study. The individual dietary diversity score (DDS) of the study respondents was calculated according to the FAO guidelines [19].

Dietary diversity was calculated as the sum of scores in each of the seven food groups, with a scale of 0 to 7. The minimum dietary diversity (MDD) indicator was calculated using at least four of the seven food groups mentioned below: (1) staples (cereals/grains, roots, and tubers); (2) dairy products; (3) animal/flesh foods; (4) legumes and nuts; (5) vitamin A-rich fruits and vegetables; (6) eggs and (7) other fruits and vegetables [19]. The Ethiopia Demographic and Health Survey factors were used to create the Household Wealth Index, which is focused on household ownership of fixed assets, services, housing characteristics, and other factors [20].

### Anthropometric measurement

Standard anthropometric procedures were used to measure the children’s height and weight [21]. The children’s heights were measured using a portable stadiometer. All of the children were told to take off their shoes and stand erect, with their heels, knees, buttocks, shoulders, and heads in contact with the stadiometer’s wall and their eyes straight ahead (Frankfurt plane) so that their line of sight was perpendicular to their bodies.

The height was measured to the nearest 0.1 cm. A portable digital scale was used to measure the weight (Seca, Germany Model). The weight was registered to the nearest 0.1 kg. It was regularly calibrated against a known weight. During the training, a standardization exercise was conducted to capture technical measurement errors before the actual anthropometric data collection (TEM). The children wore light clothes and removed their shoes for the procedure. Height for age Z-scores (HAZ), weight for height Z-scores (WHZ), and weight for age Z-scores (WAZ) were calculated using an anthropometric calculator. Moderately stunted, wasted, and underweight children had Z-scores of − 3 to − 2 SD in HAZ, WHZ, and WAZ [22].

The composite index of anthropometric failure was used to calculate the overall prevalence of under-nutrition in pre-school children (CIAF). The Nandy et al. model was used to divide CIAF into seven (7) groups. Group A (no failure), group B (waste only), group C (waste and underweight), group D (stunting, wasting, and underweight), group E (stunting and underweight), group F stunting only), and group Y (underweight only) (Tables 1), [23].

**Table 1:** classification of composite index of anthropometric failure (CIAF)*

Group	Description	Stunting	Wasting	Underweight
A	No failure	No	No	No
B	Wasting only	No	Yes	No
C	Wasting and underweight	No	Yes	Yes
D	Stunting, wasting and underweight	Yes	Yes	Yes
E	Stunting and underweight	Yes	No	Yes
F	Stunting only	Yes	No	No
Y	Underweight only	No	No	Yes

*adapted from Nandy et al., 2005

### Data processing and analysis

Data was checked, cleaned, coded and entered into Epi-data 3.1 version and then it was exported to SPSS (version 21.0) for further analysis. The baseline difference in demographic and socio-economic characteristics between the two groups was examined using a chi-square test. A t-test was used to determine the mean difference in under-nutrition between the intervention and control groups. Generalized estimating equations (GEE) with a binary logistic function and exchangeable correlation structure, were used to determine the change in the difference in outcome between the intervention and control groups as well as the association of predictors with under-nutrition in children. Accordingly, the bi-variable GEE for socio- demographic and economic factors, water and hygiene habits, maternal and child health, and child feeding habits factors for child under-nutrition were fitted. All variables in the bivariable with a *p*-value of 0.25 were fitted into a multivariable GEE. Time and treatment interaction were used to determine the intervention’s effectiveness.

The Adjusted odds ratio (AOR) with the corresponding 95% confidence intervals was reported to show the strength of the association. All analyses were conducted with the goal of considering the (ITT) concept. Variables with a p-value of less than 0.05 were considered statistically significant in multivariable analysis.

For anthropometric data analysis, anthropometric indices were calculated by WHO Anthro software 3.2.2 using WHO child growth references. The z-scores of (< −2SD) were calculated to determine HAZ, WHZ and WAZ category of stunting, wasting and underweight, respectively. Finally, CIAF was computed from the above three anthropometric indices. Any child who has one of the six different types of anthropometric measurements is classified as having CIAF.

## Result

Prior to the implementation of nutritional education, there was no statistically significant difference between the two groups were observed in all variables except, ANC follow up and had ARI, where there was a significant difference (*P* < 0.025) and (*P* < 0.0001) between the intervention and control groups, respectively. In all demographic variables, both groups had a similar distribution. The mean (+SD) age of the child in months and caregivers/mothers in year were 36.785 (+ 9.288) and 28.415 (+ 5.88) respectively. The majority of caregivers/mothers, 484 (85.1%), were married, and only 27 (4.7%) had a tertiary education **(**Table 2**).**

**Table 2:** Baseline demographic and socio-economic characteristics of participant in Southwest Ethiopia, 2019–2020

Variables	Category	Intervention(%),n=	Control (%),n=	P-value
**Age of mother/ caregiver**	< 25	104 (36.7)	97 (33.9)	0.695
	25–34	130 (45.9)	133 (46.5)	
	> = 35	49 (17.3)	56 (19.6)	
**Marital status**	Single	23 (8.1)	17 (5.9)	0.685
	Married	239 (84.5)	245 (85.7)	
	Widowed	7 (2.5)	10 (3.5)	
	Divorced	14 (4.9)	14 (4.9)	
	Divorced			
**Educational status of mothers/caregiver**	Can’t read and write	50 (17.7)	41 (14.3)	0.621
	Can read and write	25 (8.8)	25 (8.7)	
	Grade 1–4	50 (17.7)	41 (14.3)	
	Grade 5–8	79 (27.9)	88 (30.8)	
	Grade 9–12	68 (24.0)	75 (26.2)	
	Tertiary education	11 (3.9)	16 (5.6)	
**Occupation of the mothers/ caregiver**	Housewife	230 (81.3)	225 (78.7)	0.631
	Merchant	33 (11.7)	32 (11.2)	
	Government employee	12 (4.2)	18 (6.3)	
	Others*	8 (2.8)	11 (3.8)	
**Family size**	< 5	220 (77.7)	212 (74.1)	0.314
	> = 5	63 (22.3)	74 (25.9)	
**Age of the child in month**	24–35	128 (45.2)	122 (42.7)	0.731
	36–47	97 (34.3)	107 (37.4)	
	48–59	58 (20.5)	57 (19.9)	
**Sex of the child**	Male	150 (53.0)	141 (49.3)	0.377
	Female	133 (47.0)	145 (50.7)	
**ANC follow up**	Yes	272 (96.1)	262 (91.6)	0.025
	No	11 (3.9)	24 (8.4)	
**Place of deliver**	Health facility	218 (77.0)	213 (74.5)	0.477
	At Home	65 (23.0)	73 (25.5)	
**Vaccination status**	Incomplete	77 (27.2)	76 (26.6)	
	Complete	206 (72.8)	210 (73.4)	
**Had diarrhea**	Yes	79 (27.9)	87 (30.4)	0.511
	No	204 (72.1)	199 (69.6)	
**Had fever**	Yes	76 (26.9)	119 (41.6)	0.0604
	No	207 (73.1)	167 (58.4)	
**Had ARI**	Yes	62 (21.9)	110 (38.5)	0.0001
	No	221 (78.1)	176 (61.5)	
**Household food security**	Food secure	98 (34.6)	101 (35.3)	0.864
	Food insecure	185 (65.4)	185 (64.7)	
**Household Wealth index**	Lowest	92 (32.5)	105 (36.7)	0.572
	Second	19 (6.7)	19 (6.6)	
	Meddle	74 (26.1)	64 (22.4)	
	Fourth	54 (19.1)	62 (21.7)	
	highest	44 (15.5)	36 (12.0)	
**Source of drinking water**	Improved water	149 (54.8)	141 (50.9)	0.363
	Non improved water	123 (45.2)	136 (49.1)	
**Sanitation**	Improved	176 (64.7)	195 (70.4)	0.154
	Non improved	96 (35.30	82 (29.6)	
**DDS**	< 4 food group	154 (54.4)	151 (52.8)	0.698
	> = 4 food group	129 (45.6)	135 (47.2)	

Other* = Daily labor, ANC = Anti Natal Care, ARI = Acute respiratory infection, DDS = Dietary diversity score

### Effect of nutritional education on conventional anthropometric indices and CIAF

End-line data indicated the magnitude of wasting; stunting underweight and CIAF in the intervention group were significantly lower than control group. At the end of the study, the proportion of children with wasting, stunting, underweight, and CIAF increased slightly in the control group. The proportion of wasting among children was decreased by 5.5% in the intervention group while it was increased by 1.6% in the control group. The average proportion of wasting difference between the two groups was 7.1%. Additionally, in the intervention group, underweight decreased by 5.7%, whereas in the control group, it showed a 2% increasing, with an overall difference of 7.7% between the two groups. In the intervention group, the proportion of children who had overall under-nutrition decreased by 8.2%, whereas in the control group it increased by 1.3%. Between the two groups, the average proportion of overall malnutrition (CIAF) was 11% **(**Table 3**).**

**Table 3:** Differences between baseline and end line anthropometric failure and differences in differences between intervention and control groups

Variable	Intervention group			Control group			Difference in difference
	Base line	End line	Difference (EL-BL)	Base Line	End Line	Difference (EL-BL)	
**Wasting**	0.124	0.069	−0.055*	0.139	0.155	0.016*	−0.071*
**Stunting**	0.456	0.393	−0.063	0.395	0.412	0.017	−0.08
**Underweight**	0.237	0.180	−0.057**	0.312	0.332	0.02*	−0.077**
**CIAF**	0.516	0.434	−0.082	0.485	0.513	0.028	−0.11

*P < 0.01, ** *P* < 0.001, EL = End Line, BL = Base Line, CIAF = Composite index of anthropometric

### Effect of the intervention on the overall under nutrition of preschool children

To identify independent factors associated with the end line-baseline difference of the differences in the mean CIAF scores, we employed multivariable Generalized Estimating Equations (GEE). Variables included in the model were: wealth index, family size, occupational status, and place of delivery, fever, ARI, source of drinking water, food security and nutritional education. Out of all other variables, nutritional education, place of delivery, ARI in the last 2 weeks, source of drinking water and food security were independent predictors of CIAF. After controlling for possible confounders preschool children in intervention group were 43.4% less likely to have CIAF compared to the control group [AOR = 0.566, 95% CI: (0.347, 0.923)]. Children born at health facility were 27.6% less likely to have CIAF compared to children born at home [AOR = 0.724, 95% CI: (0.551, 0.951)]. Children who had ARI in the last 2 weeks were 1.823 more likely to develop CIAF than children who hadn’t ARI [AOR = 1.823, 95% CI: (1.226, 2.710)]. Preschool children from household who used improved source of drinking water were 37.6% less likely have CIAF compared to household used non improved source of drinking water [AOR = 0.624, 95% CI: (0.484, 0.805)].Children from food insecure household were [AOR = 1.311, 95% CI: (1.030, 1.669)] **(**Table 4**).**

**Table 4:** Generalized estimating equations model for among preschool aged children in south west Ethiopia, 2019–2020

Predictors	Composite index of anthropometric failure					
	B	SE	P	AOR	95% Confidence interval	
					Lower	Upper
**Nutritional Education**						
**Time**	0.478	0.1771	0.007	1.613	1.14	2.283
**Group**	0.166	0.1767	0.346	1.181	0.835	1.67
Time *Group	−0.569	0.2494	0.023	0.566	0.347	0.923
**Family size (Ref = < 5)**						
**> =5**	0.008	0.1341	0.952	1.008	0.775	1.311
**Wealth index (Ref = lowest)**						
**Second**	−0.444	0.2663	0.096	0.642	0.381	1.081
**Meddle**	0.227	0.1697	0.180	1.255	0.900	1.751
**Fourth**	0.105	0.1724	0.544	1.11	0.792	1.557
**highest**	0.301	0.1972	0.127	1.351	0.918	1.988
**Occupational status (Ref = Housewife)**						
**Merchant**	0.050	0.1795	0.781	1.051	0.739	1.494
**Government employee**	0.259	0.3044	0.395	1.295	0.713	2.353
**Others**	−0.525	0.3578	0.143	0.592	0.918	1.988
**Place of deliver (Ref = At health facility)**						
**At home**	−0.323	0.1392	0.023	0.724	0.551	0.951
**Fever (Ref = Yes)**						
**No**	−0.127	0.1796	0.480	0.881	0.619	1.253
**ARI in the last 2 weeks (Ref = Yes)**						
**No**	−0.600	0.2024	0.003	1.823	1.226	2.710
**Source of drinking water (Ref = Improved)**						
**Non improved**	−0.472	0.1299	0.001	0.624	0.484	0.805
**Food security Ref = Secured)**						
**In secured**	0.271	0.1232	0.028	1.311	1.030	1.669

B=Beta, SE = Standard Error, P = P-value, AOR = Adjusted Odd Ratio, Ref = Reference category

## Discussion

The purpose of this community-based quasi-experiment was to investigate the effectiveness of a nutrition education intervention delivered by trained health professionals on the anthropometric failures of preschool-aged children. The nine-month nutrition education program aims to lower preschool-aged children’s wasting, stunting, and underweight, as well as overall under-nutrition.

The intervention’s major goal was to encourage the consumption of a diverse variety of locally accessible and affordable nutritious foods in order to increase the adequacy of traditional home-cooked diets as well as environmental hygiene and sanitation. The intervention had a significant impact on the children’s nutritional status. This implies that a nutrition education intervention without the use of food supplements can help preschool-aged children to improve their nutritional status.

There was no significant difference in nutritional status between the intervention and control groups at the start of the study. However, after nine months of nutrition education intervention, there was a significant difference between the intervention and control groups for wasting and underweight, while improvements in stunting and overall under-nutrition (CIAF) were also found, but the difference was not significant, as shown in Table 3. This finding is in line with the study conducted in Burkina Faso among preschool children in which the anthropometric indices showed significant changes after intervention [24]. The findings of a systematic review on community-based nutrition education in developing countries are similar to those of the current study [14].

An interventional study conducted in Indonesia is similar to the current study in that they have a significant effect on underweight and an insignificant effect on stunting, but they are inconsistent in that they have an insignificant effect on wasting [25]. The possible reason for the discrepancy could be the duration of intervention, which in the current study, the intervention period was nine months, while in the previous study; the duration of intervention was five months. This is because the short-term intervention was insufficient to improve the nutritional status of children who were stunted in particular. Height-for-age was an effect of long-term food consumption, and the intervention’s short duration was insufficient to improve it [25]. In line with the current study, results from an interventional study conducted in Ethiopia and Tanzania [26, 27] indicated no significant change in child stunting. The result of a nutritional education intervention conducted in Limpopo province, South Africa, contradicts the current study because no anthropometric indices changed significantly after the intervention [28]. Different intervention strategies and intensities, differences in the age of the children upon enrolling, and pre-existing children’s growth and nutritional status might be possible reasons for the discrepancy.

In this study, the average proportion of wasting, stunting and underweight differences between the two groups was 7.1, 8 and 7.7%, respectively. Between the two groups, the average proportion of overall malnutrition (CIAF) was 11%. The current study is also consistent with the Alexandria study, which found a decrease in the percentage of underweight, stunted, and wasted people [29]. The nutrition intervention conducted in Bhopal district, Madhya Pradesh showed a percentage reduction in underweight among children after intervention [30].

After controlling for possible confounders, multivariable analysis revealed that factors such as nutritional education, place of delivery, ARI in the previous two weeks, source of drinking water, and household food security were statistically significant with overall under-nutrition in preschool children. The current study showed that the odds of being under-nourished (CIAF) were less likely to occur among children in the intervention group compared to children in the control group. The findings of the current study are in line with a quasi-experimental study conducted in Chandigarh, India in which intervention had a significant effect on reduction of under-nutrition [31].

The result of this study showed that the place of delivery of the mother is a significant predictor of the nutritional status of children. Children whose mothers had home delivery were at a higher risk of being under-nourished than children whose mothers had health facility delivery. The findings were comparable with the study conducted in Ethiopia [32]. The findings of the study indicated that children who had ARI in the last two weeks were more likely to develop under-nutrition (CIAF) than those who did not. This finding was corroborated by the study conducted in Burkina Faso and urban slums of Agra city in which the ARI was significantly associated with child under-nutrition [33, 34].

The study found that the source of drinking water was a significant factor of under-nutrition in which children from families who had an unimproved water source were more likely to be under-nourished compared with children from families who had an improved source of water. The finding was consistent with different studies conducted in Ethiopia and India [33, 35–37]. This study showed that children from food-insecure households are at a higher risk of under-nutrition. Similar findings from west Oromia of Ethiopia indicate that household food insecurity is significantly associated with the under-nutrition of children [38].

In general, the findings of this research indicate the effectiveness of nutrition education in reducing the average proportion of wasting, stunting, underweight, and overall malnutrition (CIAF). A number of methodological issues affected this study, many of which were beyond the project’s control. The data collected from the mothers/caregivers was based on recall over a prolonged period of time, which could lead to memory bias, particularly for infant feeding practices and other retrospective data relying on the mother’s remember of previous events. This data could have been distorted by a social-desirability bias, which resulted in the over-reporting of good behaviors. Another limitation could be our inability to determine mothers’ nutritional status. Finally, we only had two data collection sessions in our study (at baseline and end line). In the future, we recommend that comparable studies include more follow-up visits and assessments to reinforce behavior change and adoption of the recommended child feeding practices.

## Conclusions

Our finding revealed that community-based nutrition educational intervention could effectively decrease the percentage of the overall under- nutrition of preschool children. This intervention had a significant reduction on children’s wasting and underweight, whereas no significant reduction in children’s stunting and CIAF. The overall under-nutrition was found to be significantly associated nutritional education, place of delivery, ARI in the last 2 weeks, source of drinking water and food security. It is recommended that children’s nutritional status in rural areas should be improved through community based nutrition education intervention. This effective means of reducing under-nutrition in children should be scaled up and adapted to other zones and the regions as preventive and management strategies and should involve the community to build culturally-based skills for long term nutrition goals. Health education should be needed to promote health facility delivery; preventing practice of infection diseases, increasing the coverage of improved source of drinking water for the rural areas and different strategies should be performed to maintain household food security. In addition, further research should be done to explore at associated factors that were not discussed in this study, as well as a future study assessing the nutritional status of mothers. Finally, these findings add to the evidence that there is room for improvement in children’s feeding behavior and growth through nutrition education and behavior change communication.

## Data Availability

The data is at presently unavailable as it part of an unending PhD Project.

## Abbreviation

WHO: World Health Organization
CIAF: Composite Index of Anthropometric failure
AOR: Adjusted Odds Ratio
COR: Crude Odds Ratio
DDS: Dietary Diversity Score
ARI: Acute Respiratory Infection
EDHS: Ethiopia Demographic Health Survey
UNICEF: United Nations Children’s Fund
ANC: Anti natal Care
MDD: Minimum Dietary Diversity
FANTA: Food and Nutritional Technical Assistance
TEM: Technical error of Measurement
HAZ: Height for Age
WHZ: Weight for Height
WAZ: Weight for Age
SD: Standard Deviation
SPSS: Statistical Package for Social Science
